# How ants send signals in saliva

**DOI:** 10.7554/eLife.23375

**Published:** 2016-12-12

**Authors:** Markus Knaden

**Affiliations:** 1 Department of Evolutionary Neuroethology, Max Planck Institute for Chemical Ecology, Jena, Germanymknaden@ice.mpg.de

**Keywords:** *Camponotus floridanus*, *Apis mellifera*, *Solenopsis invicta*, *Camponotus fellah*, social evolution, collective behavior, Other

## Abstract

Adult ants use saliva to transfer juvenile hormone and other chemical signals to their larvae.

**Related research article** LeBoeuf AC, Waridel P, Brent CS, Gonçalves AN, Menin L, Ortiz D, Riba-Grognuz O, Koto A, Soares ZG, Privman E, Miska EA, Benton R, Keller L. 2016. Oral transfer of chemical cues, growth proteins and hormones in social insects. *eLife* **5**:e20375. doi: 10.7554/eLife.20375

Ant colonies are complex systems in which each ant fulfills a specific role to help the whole colony survive. The ants in a colony develop into distinct types known as castes to perform these roles. In colonies of leaf cutter ants, for example, small “worker” ants usually care for the larvae and the fungus the colony feeds on, while larger worker ants leave the nest to forage for new leaves to grow the fungus on. Other species, such as the silver ant, possess a soldier caste that has huge mouthparts dedicated to fighting. Finally, most colonies have one or several “queen” ants that focus on reproduction. It is important that the colony has the right numbers of each caste: if too many ants develop into soldiers, for example, the colony will starve, while a colony with too many foragers cannot take care of its larvae.

Genetic cues, environmental cues like food or the size of the colony, or a combination of both, can determine the caste that an individual will become (for a review see Schwander et al., 2010). Adult ants are able to influence the caste fate of larvae by changing the types of food they provide, by producing chemicals known as pheromones, and by regulating the temperature of the chamber the larvae live in (Wheeler, 1991). Now, in eLife, Laurent Keller and Richard Benton of the University of Lausanne and colleagues – including Adria LeBoeuf as first author – report on a new way in which adult ants can alter how larvae develop (LeBoeuf et al., 2016).

Juvenile hormone regulates development and reproduction in insects and also appears to affect caste fate in ants (Wheeler, 1986; Wheeler and Nijhout, 1981; Rajakumar et al., 2012; Nijhout, 1994). When the larvae of an ant called *Pheidole bicarinata* were exposed to increased amounts of a molecule that is very similar to juvenile hormone, most of them became soldiers instead of workers (Wheeler and Nijhout, 1981). However, it was not clear how the levels of this hormone were regulated in larvae.

Like most other social insects, adult ants feed their larvae by transferring fluid (saliva) mouth-to-mouth in a process called trophallaxis. LeBoeuf et al. – who are based at institutes in Switzerland, the US, Brazil, Japan and the UK – used mass spectroscopy and RNA sequencing to identify the molecules present in the saliva of the Florida carpenter ant (*Camponotus floridanus*). They found that, in addition to nutrients, ant saliva also contains juvenile hormone and other molecules including proteins, microRNAs and cuticular hydrocarbons (Figure 1). Furthermore, the amount of juvenile hormone transferred by trophallaxis is high enough to affect how the larvae develop.

**Figure 1.**
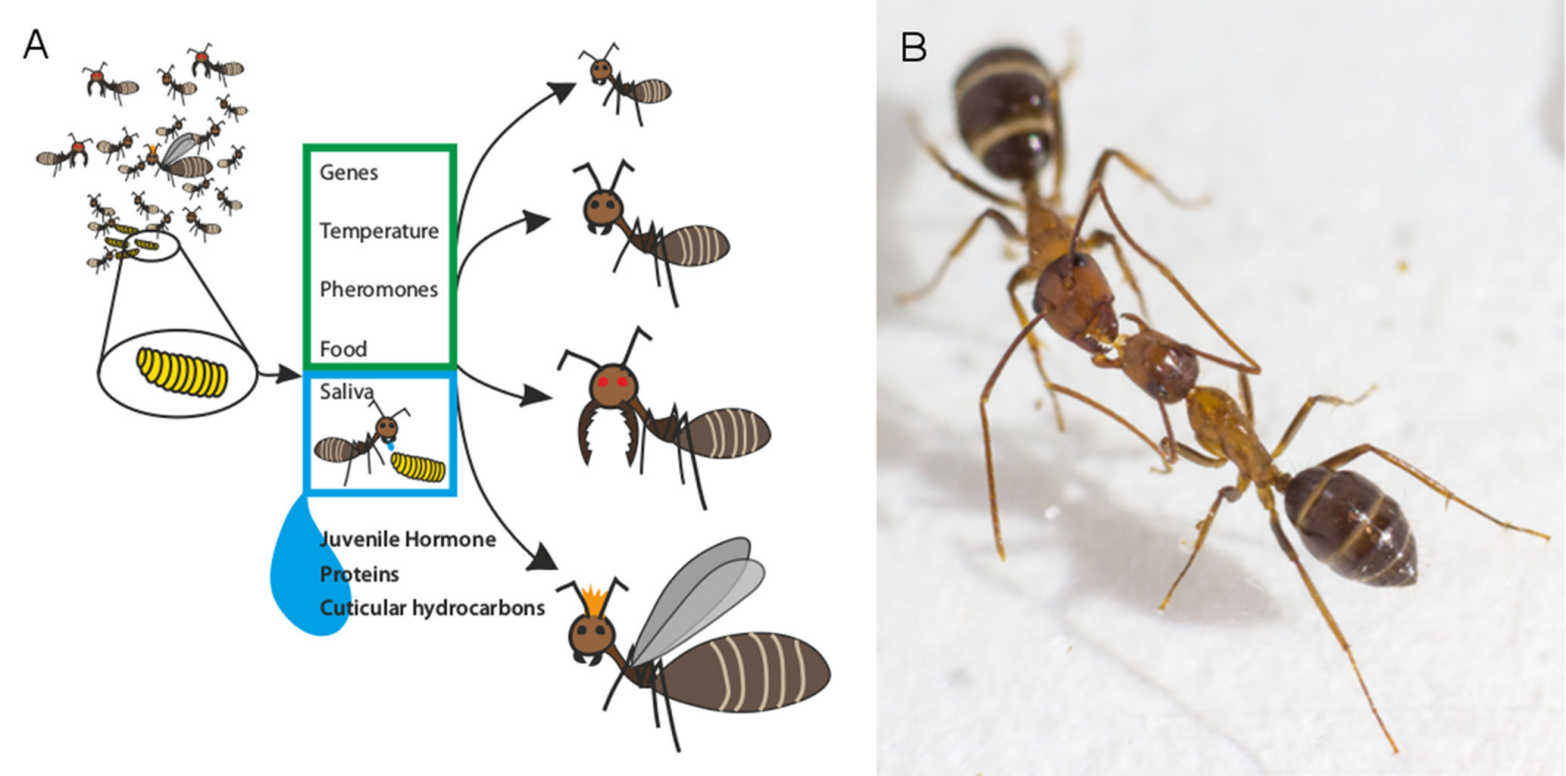
Several factors influence the roles of individual ants in ant colonies. (**A**) As a larva (yellow) prepares to transform into an adult ant, a number of factors determine whether it will develop into, for example, a nurse, a forager, a soldier or a queen (right: top-to-bottom). Previous studies have shown that genetic factors and environmental cues can influence this outcome (green box). LeBoeuf et al. show that when adult ants feed larvae mouth-to-mouth (blue box), a variety of signal molecules are transferred alongside the nutrients in the saliva of the adult. These molecules include juvenile hormone, which is known to alter caste fate, and numerous proteins that control how social insects grow and develop. Molecules known as cuticular hydrocarbons (which allow ants to distinguish between nestmates and non-nestmates) are also transferred. (**B**) Adult ants also exchange fluid mouth-to-mouth, as demonstrated in this photograph (taken by LeBoeuf et al.) of two carpenter ants.

Alongside juvenile hormone, some other molecules in the saliva may also be acting as chemical signals: for example, it is known that cuticular hydrocarbons help ants to discriminate nestmates from non-nestmates (Hefetz, 2007). Furthermore, many of the proteins LeBeouf et al. identified in carpenter ants are involved in regulating the growth, development and behavior of other social insects.

This study is the first to show that trophallaxis can circulate juvenile hormone and other proteins that might be involved in larval development and caste fate around the colony. It also suggests that the ants exchange other hormones that we did not previously know were involved in communication between individuals: these hormones include hexamerin, which is known to be involved in caste fate in social insects (Zhou et al., 2007). The next challenge will be to find out whether trophallaxis really plays an active role in regulating caste fate. One way to test this idea would be to remove all the soldiers from the colony and observe whether this changes the amount of different hormones present in ant saliva as the colony attempts to replace the soldiers. Adult ants are best placed to know the needs of the colony, so it makes sense that they use several strategies to guarantee that the colony produces the right mix of castes.
